# Divergent Evolution of Tuberculosis Lesions During Treatment: A Longitudinal CT-Based Analysis of Progression and Regression Patterns

**DOI:** 10.3390/diagnostics16060892

**Published:** 2026-03-18

**Authors:** Liyi Qin, Jiaxin Jiang, Shiran Ma, Xiaoming Liu, Pingxin Lv, Wei Wang, Howard E. Takiff, Yingda L. Xie, Qingyun Liu, Weimin Li

**Affiliations:** 1 Beijing Chest Hospital, Capital Medical University, Beijing 101149, China; raven_million@163.com (L.Q.); shiranma@163.com (S.M.); liuxiaoming313@163.com (X.L.); wangwei010@aliyun.com (W.W.); 2 Beijing Tuberculosis and Thoracic Tumor Research Institute, Beijing 101149, China; 3 National Clinical Research Center for Cardiovascular Diseases, Fuwai Hospital, Chinese Academy of Medical Sciences, Beijing 100037, China; 4 Department of Biostatistics, New York University, New York, NY 10032, USA; gloriajiang1122@gmail.com; 5 Department of Radiology, Being Geriatric Hospital, Beijing 100095, China; lpx1209@163.com; 6 Beijing Key Laboratory for Drug-Resistant Tuberculosis Research, Beijing Chest Hospital, Capital Medical University, Beijing 101149, China; 7 Beijing Municipal Key Laboratory of Clinical Epidemiology, School of Public Health, Capital Medical University, Beijing 101149, China; 8 Centro de Microbiología y Biología Celular, Instituto Venezolano de Investigaciones Científicas (IVIC), Caracas 1020, Venezuela; htakiff@gmail.com; 9 Department of Medicine, Public Health Research Institute, Rutgers New Jersey Medical School, Newark, NJ 07103, USA; 10 Department of Genetics, University of North Carolina at Chapel Hill, Chapel Hill, NC 27599, USA; 11 Department of Microbiology and Immunology, UNC Chapel Hill School of Medicine, Chapel Hill, NC 27599, USA

**Keywords:** tuberculosis, computed tomography, lesion heterogeneity, personalized medicine

## Abstract

**Objectives:** Lesion-level dynamics may reveal pulmonary tuberculosis (PTB) heterogeneity and help identify factors associated with treatment outcomes. **Methods:** A total of 288 serial Computed Tomography (CT) scans from 125 PTB patients were obtained from the National Institute of Allergy and Infectious Diseases (NIAID) TB Portals database (2008–2023). Lesions were segmented and annotated to obtain volume and imaging features, and a conservative longitudinal volume quantification method was used to characterize dynamic volume patterns. The proportion of lesions with different patterns was analyzed at the patient level to assess trajectory diversity. Firth’s penalized logistic regression was used to identify factors associated with treatment outcomes. **Results:** Among 435 lesions in 125 patients, five patterns emerged: Stable, Decrease, Increase, Mix-I-D (increase then decrease), and Mix-D-I (decrease then increase). Multiple patterns coexisted in 66.7% of treatment success patients and all treatment failure patients. Mix-D-I lesions were identified more frequently in treatment failure patients (25.0% vs. 1.4%, *p* = 0.027), and in multivariable analysis, the presence of Mix-D-I lesions was statistically associated with treatment failure (*p* = 0.024). **Conclusions:** PTB lesions showed high trajectory heterogeneity. The presence of Mix-D-I lesions may point to an unfavorable treatment course, suggesting lesion dynamics could serve as a potential indicator for poor outcomes. By quantifying lesion-level trajectories on serial CT scans, we extend PET/CT-based evidence and support the value of routine monitoring in clinical management of tuberculosis.

## 1. Introduction

Tuberculosis (TB), caused by *Mycobacterium tuberculosis* (*Mtb*), remains the leading infectious cause of death worldwide [1]. One of the major obstacles to curing *Mtb* is the formation of multiple spatially independent lung lesions, each of which can contain distinct microenvironments where bacterial persistence or clearance is determined by the local drug concentrations and host–pathogen interactions [2,3,4,5]. Even within a single host, individual lesions can exhibit disparate evolutionary trajectories, ranging from sterile resolution to progressive cavitation [6,7,8]. While the heterogeneity of different infectious foci has been well documented, few studies have examined how their disparate evolution affects and predicts treatment outcomes.

Advanced imaging modalities have enhanced our capacity to map this spatiotemporal complexity. While positron emission tomography with computed tomography (PET/CT) provides functional–metabolic insights in non-human primate models [6,9,10], conventional CT is more appropriate for clinical monitoring due to its superior resolution, widespread availability and lower cost [11]. Although most studies have focused on whole-lung analyses of total lesion burden, a growing body of evidence highlights the heterogeneity of lesions in patients with pulmonary TB (PTB). The Catalysis treatment response cohort—the largest prospective PET/CT study in humans—revealed that over one-third of PTB patients exhibit concurrent lesion progression and regression during therapy [12,13,14]. Furthermore, lesion dynamics in PTB patients may have important implications for treatment outcomes. Recent studies have shown that dynamic changes in the total lesion burden are more strongly correlated with clinical outcomes than baseline measurements alone [12,15]. Additionally, emerging evidence suggests that lesions with fluctuating dynamics may harbor heteroresistant *Mtb* subpopulations that can contribute to treatment failure [16,17]. However, evidence on lesion-level dynamics from routine clinical CT remains limited. To provide insights on the significance of lesion evolution, we performed a detailed investigation of individual lesion trajectories and their ability to predict treatment outcomes in human PTB.

The National Institute of Allergy and Infectious Diseases (NIAID) TB Portals database (2008–2023) is an open-access repository that integrates multi-domain data from over 10 high-burden TB countries and contains sociodemographic, clinical, imaging, and pathogen information [18,19]. From the database, 125 patients with available longitudinal CT scans were included for quantitative tracking of lesion dynamics. We hypothesized that lesions within PTB patients show substantial heterogeneity in evolutionary trajectories and that specific dynamic patterns are associated with poor treatment outcomes.

## 2. Methods

### 2.1. Patients and CT Series

The NIAID TB Portals Program (https://tbportals.niaid.nih.gov/) is an open-access repository that aggregates de-identified clinical metadata from tuberculosis patients contributed by multiple institutions across high-burden settings. These data were collected in different clinical and research contexts under varying acquisition protocols; CT scans were obtained as part of routine clinical care.

**Figure 1:**
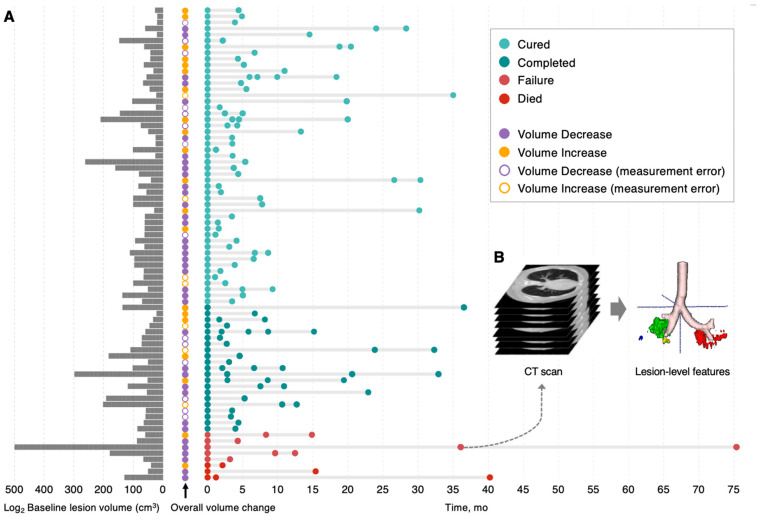
Study population characteristics and data processing for lesion-level quantitative tracking. (**A**) Temporal distribution of 185 serial CT scans from 78 PTB patients with different treatment outcomes. Light and dark turquoise indicate CTs from treatment success patients; red and maroon indicate CTs from treatment failure patients. The log2 baseline whole-lung involvement volume is displayed for each patient. Overall volume change demonstrates the variation in total lesion volume between initial and final CT scans, where open circles indicate patients without significant volume changes and differences attributable to measurement error. Solid yellow circles denote substantial enlargement, and lavender circles denote reduction. (**B**) Lesions from each CT scan were independently segmented to extract lesion-level characteristics, including volumetric measurements, morphological features, and dynamic evolution patterns. The 3D reconstruction model uses pale pink to represent bronchial structures and distinct colors to represent independent lesions. The dashed arrow indicates that lesion-level features were extracted from CT scans.

Out of the total 11,067 patients in the NIAID TB Portals database (2008 and 2023), 125 PTB patients with recorded treatment outcomes of “cured” or “completed” (classified as “treatment success”), and “failure” or “died” (classified as “treatment failure”) were included in this longitudinal retrospective study. We collected demographic and clinical data from these 125 patients, including all available serial CT images. Eligibility required at least two chest CT scans taken more than 1 month apart. After removing duplicate CT scans, there was a total of 288 CT studies, and for each of these CT scans, all lesions were independently tracked and analyzed (Figure 1A,B, Supplementary Figure S1, Supplementary Table S4).

### 2.2. CT Segmentation and Image Annotations

For patients with widespread parenchymal involvement and poorly defined lesions, precise lesion-level quantification was not feasible and therefore these lesions were assessed visually and described at the lobar level. For the remaining patients, all pulmonary parenchymal lesions ≥ 3 mm on CT were segmented using ITK-Snap (Version 3.6.0). We delineated each lesion layer by layer, generating a three-dimensional volume of interest (VOI). To account for bronchogenic spread and the frequent fusion of pulmonary lesions, we established independent lesion delineation criteria to ensure segmentation consistency (Figure 1B, Supplementary Text S1, Supplementary Figure S2A–E). These criteria emphasized bronchial drainage areas, whereby spatially adjacent yet discontinuous lesions could be grouped into a single VOI, facilitating lesion alignment across multiple time points. Specifically, on each patient’s initial CT scan, the lesions were numbered independently, and each lesion’s VOI was then sequentially aligned to VOIs in subsequent scans based on spatial proximity. VOIs occupying similar locations shared the same lesion number, while unmatched VOIs were assigned new identifiers. In cases where a single lesion split into multiple VOIs, any subsequent VOI extending clearly beyond the original lesion boundary was classified as a new lesion. This alignment procedure effectively preserved lesion progression information and simplified lesion tracking.

To characterize lesion morphology and activity, each VOI was annotated with three imaging feature categories: (1) primary lesion type, defining the main lesion’s morphology; (2) satellite lesion type, describing surrounding lesions; and (3) accompanying characteristics, based on density and activity, classified as “Slight to Moderate Density”, “Cavity”, “Fibrosis”, or “Calcification”, with the first two regarded as “Active” lesions (Supplementary Text S2, Supplementary Figure S2A–E).

Two experienced chest clinicians, blinded to treatment outcomes, independently assessed, segmented and annotated all lesions. Any discrepancies were resolved through discussion until consensus was reached.

### 2.3. Longitudinal Tracking of Lesion Volume

To conservatively detect significant longitudinal changes in lesion volume, we developed an estimation scheme based on principles consistent with previous sphere approximation and threshold methods [20,21]. This approach corrects for potential measurement errors introduced by variability in CT acquisition (e.g., differences in lung expansion between scans), manual segmentation inconsistencies and lesion alignment across time points [22,23,24] (details in Supplementary Text S3, Supplementary Figure S3A–C). The error for each lesion was defined as the spatial range resulting from expanding or contracting its VOI by one voxel (the smallest volumetric unit in CT imaging) in all directions. Given that VOIs consist of irregular voxel stacks, direct calculation of volume error is complex. To simplify, we approximated each VOI as a sphere with an equivalent volume and estimated the error by computing the volume change resulting from a slight diameter adjustment (Supplementary Figure S3A). This geometric simplification acknowledges the complex three-dimensional morphology of tuberculous lesions [2]. With this framework, small lesions were more affected by minor changes in the diameter than larger ones, and therefore we set multiple volume thresholds: if the volume difference between two consecutive measurements of the same lesion fell within the threshold, the change was attributed to measurement error and considered stable. If the volume difference between consecutive CT scans exceeded the threshold, the lesion was classified as having significantly increased or decreased in volume (Supplementary Figure S3B,C). We applied the same method to characterize lesions that occupied the whole lung by comparing the initial and final scans and using the baseline total lung volume to evaluate disease progression.

This estimation method allowed us to track lesion dynamics. The volume of each lesion between two consecutive CT scans was classified as increased, decreased or stable, with stability defined as volume changes within the measurement error range. Because some patients had multiple serial CT scans, with fluctuating changes in lesion volume, we defined five longitudinal volume patterns:

(1) Stable: Lesion volume remained consistent across all scans, with no significant enlargement or reduction, and all variations were attributed to measurement error.
(2) Decrease: Lesions showing a significant reduction in volume between two CT scans. For lesions with three or more scans, there was an overall decline in volume that could include stable periods but no instances of volume increase.
(3) Increase: Lesions showing a significant increase in volume between two CT scans. For lesions with three or more scans, an overall enlargement was observed that could include stable periods but no reduction in volume. This category also included new lesions appearing after the initial CT scan that either enlarged or remained stable without shrinking.
(4) Mix-I-D: A fluctuating pattern observed only in lesions with more than two scans, in which the lesion volume first increased and then decreased. Periods of stability between some CT scans were permitted.
(5) Mix-D-I: A fluctuating pattern observed only in lesions with more than two scans, in which the lesion volume first decreased and then increased. Periods of stability between some CT scans were permitted.

### 2.4. Statistical Analyses

We classified lesion parameters into static features—those seen on the initial CT scan, and dynamic features—changes in the lesion on subsequent CT scans (Table 1, Supplementary Table S2 and Supplementary Text S4). Continuous variables are presented as median and interquartile ranges (IQRs) or means with standard deviations (SDs), and categorical variables are shown as frequencies and percentages. Differences between groups were analyzed using the *t*-test or the Wilcoxon rank-sum test for continuous variables, and Pearson’s χ^2^ or Fisher’s exact tests for categorical variables. To reduce potential bias resulting from a low incidence of events (volume changes) and therefore small sample size, we used Firth’s penalized likelihood logistic regression to identify determinants of treatment outcomes and estimate odds ratios (OR) and 95% confidence intervals (CI) [25,26]. Univariate analysis was performed for all variables, and those meeting *p* < 0.1 were included in the multivariate model. All tests were two-sided, with statistical significance set at *p* < 0.05, and all analyses were performed in R (version 4.3.3).

### 2.5. Patient and Public Involvement

Patients or the public were not involved in the design, or conduct, or reporting, or dissemination plans of our research.

## 3. Results

### 3.1. Patients, CT Scans, and Lesion Capture

To investigate lesion evolution in PTB, 2507 CT scans were retrieved from the TB Portals database. These CT scans were originally collected from 1456 patients across nine countries between 2008 and 2023, with 68.5% originating from Belarus (Supplementary Table S1). After removing duplicate CT scans and excluding patients with a single CT scan, 134 patients remained. Further exclusion of 9 patients whose CTs were taken <1 month apart resulted in a final cohort of 125 patients with 288 serial CTs. Among these, 89.6% (112/125) achieved treatment success (Supplementary Figure S1, Supplementary Table S1). Most patients (99/125, 79.2%) had two CT scans, including both patients with treatment success and patients with treatment failure (91/112 vs. 8/13, *p* = 0.195). Additionally, there were 18, 5, and 2 patients with three, four, and five CT scans, respectively. The median intervals between sequential scans were 3.7 months (IQR: 2.4–6.7) between the first and second scans, 4.1 months (IQR: 2.1–6.3) between the second and third, 5.0 months (IQR: 2.8–11.2) between the third and fourth, and 6.5 months (IQR: 4.7–7.5) between the fourth and fifth scans. Only one patient had six CT scans, with an interval of 1.8 months between the fifth and sixth CT scans.

Of the 125 patients, 47 were excluded from quantitative tracking due to poorly defined lesion boundaries (Supplementary Figure S1), and therefore the trajectories of their 201 lesions were visually assessed at the lobar level (Supplementary Table S4). In the remaining 78 patients with 185 CT scans, the lesions were clearly segmented. These patients had a median age of 42 years and 55.1% were male. No significant differences in demographics, clinical factors, or CT scan frequency were observed between patients who experienced treatment failure and those with treatment success (Table 1, Supplementary Table S2).

A total of 234 independent lesions were tracked in these 78 patients: 198 lesions were present at baseline; 9 emerged during treatment; and 27 appeared in the final CT scans. Of the total 234 tracked lesions, 202 (86.3%) were in 70 patients with treatment success.

### 3.2. Disparate Evolutionary Trajectories of Different Lesions Within PTB Patients

Before tracking lesion-level changes, we compared whole-lung lesion volumes between initial and final scans. The lesion burden was stable or decreased in most patients (58/78, 74.4%), including 52 (52/58, 89.7%) with successful treatment, while 18 of these 58 (18/58, 31.0%) had paradoxical enlargement during treatment. Among the 8 patients with treatment failure, the lesion volume increased in 2 patients and decreased in 6 (Figure 1A).

**Figure 2:**
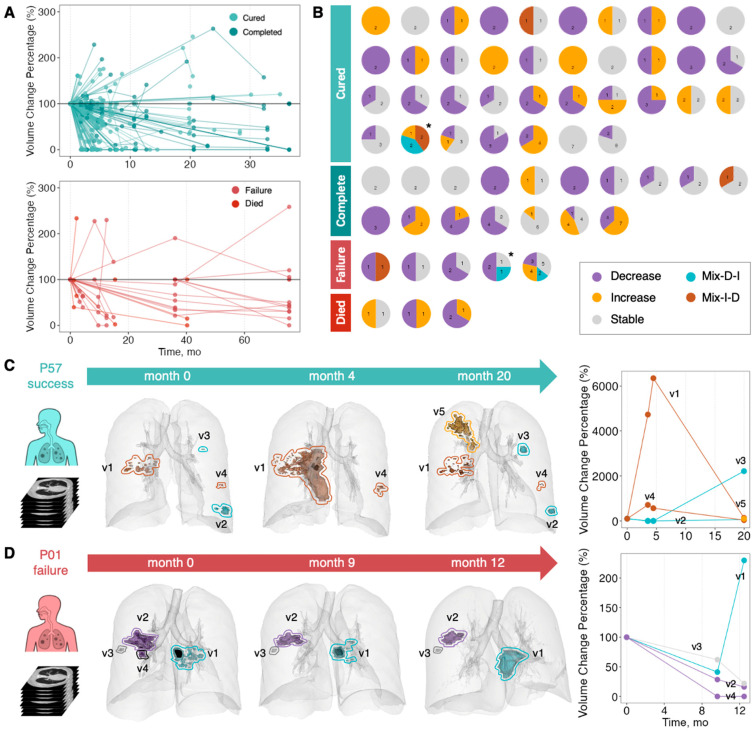
Disparate evolutionary trajectories of different lesions within PTB patients. (**A**) The line graph shows the percentage of volume change over time for lesions in PTB patients with different treatment outcomes. The initial lesion volume was set as 100%. Upper panel, lesions from patients with treatment success. Lower panel, lesions from patients with treatment failure. (**B**) The pie charts illustrate the different volume change patterns lesions within each patient, with numbers indicating lesion count. The asterisk (*) denotes representative patients P57 (success) and P01 (failure), selected for detailed visualization. (**C**) Three-dimensional reconstructions of lung lesions for patient P57 (v1–v5) and (**D**) three-dimensional reconstructions of lung lesions for patient P01 (v1–v4). These panels illustrate the evolutionary trajectories of each lesion over time. Different lesions are separated by closed lines, and both the lesion volume and the lines are color-coded according to their volume change pattern. The black regions represent the formation of cavities within the lesions. The line graph shows the percentage of volume change over time for each lesion.

At the individual lesion level, we identified five dynamic patterns across the 234 total lesions: Decreased (85 lesions); Stable (84); Increased (55); Mix-I-D—initially increased and subsequently decreased (5); and Mix-D-I—initially decreased and subsequently increased (5). The most common lesion patterns in patients achieving treatment success (202) compared to patients with treatment failure (32) were: Decrease (36.1% vs. 37.5%); Stable (37.1% vs. 28.1%); and Increase (23.8% vs. 21.9%). Both groups contained a small proportion of Mix-I-D and Mix-D-I lesions. Thus, the distribution of lesion patterns was similar in patients who were cured and those who failed treatment (Figure 2A,B).

To examine the diversity of lesion trajectories within patients, we further analyzed 62 PTB patients (54 treatment success, 8 failure) with multiple lesions. Among the 54 patients with treatment success, 29 (53.7%) had lesions with Decrease and/or Stable patterns, 8 (14.8%) had Increase lesions (with or without accompanying Stable lesions); and 3 (5.6%) had Mix-I-D lesions; 1 had Mix-D-I. A notable heterogeneity was seen in different lesions within individual patients: 66.7% (36/54) of treatment success patients exhibited two or more distinct lesion patterns, with 25.9% (14/54) having both Decrease and Increase lesions (Figure 2B). For example, patient P57 had five lesions (v1–v5) that displayed three different patterns (Figure 2C): (1) Mix-I-D lesions (v1 and v4)—v1 worsened significantly by month 4, evolving from a cluster of nodules into a cavitary consolidation with tree-in-bud spread, but showed substantial resolution by month 20, while v4, initially a cluster of nodules, increased in volume by 613.6% before shrinking by 94.6%; (2) Mix-D-I lesions (v2 and v3)—both lesions were absent at month 4 but reappeared on the last CT scan; (3) Increase lesion (v5)—a newly emerged consolidation with satellite nodules was detected only on the last CT scan.

All eight treatment failure patients exhibited multiple coexisting volume change patterns. Of these eight patients, two had Decrease + Stable lesions, one had Increase + Stable, and two had Decrease + Increase. The remaining three patients showed fluctuating patterns across multiple lesions (Figure 2B). For example, patient P01 had four distinct lesions (v1–v4) (Figure 2D): (1) Mix-D-I lesion (v1)—a cavitary nodule with tree-in-bud satellite signs shrank by month 9 but progressed to consolidation by month 12; (2) Decrease lesions (v2 and v4)—v2, a cavitary consolidation gradually fibrosed and closed, and v4, a cavitary nodule, disappeared completely by month 9; (3) Stable lesion (v3)—a calcified nodule remained unchanged.

In addition to the 78 patients included in the quantitative analysis, there were an additional 47 patients in the cohort whose lesions could not be quantitatively assessed. Among these, 37 had multiple lesions. Visual evaluation in this subgroup identified coexisting lesion patterns in 45.9% (17/37) of treatment success cases and in all treatment failure cases (Supplementary Table S4, Supplementary Figure S4).

To determine whether evolutionary heterogeneity was exclusive to patients with multiple lesions, we analyzed 16 patients with a single lesion, all from the treatment success group. Among these lesions, five showed Decrease, five remained Stable, and six showed Increase. Although an increase in lesion volume on CT scans does not directly indicate progression, we also observed the emergence of tree-in-bud satellite lesions around cavitary nodules, suggesting progression with bronchogenic spread. This shows that lesion heterogeneity is common and transient local progression or inflammatory remodeling can occur even in patients who are successfully treated.

Further analysis revealed that the initial characteristics of a lesion often predicted its subsequent evolution. Stable lesions were predominantly small, isolated nodules lacking consolidation and exhibiting fewer tree-in-bud satellites, suggesting less bronchogenic spread. Stable lesions also showed more calcification and fewer cavities. Decrease lesions were the largest, with more consolidation, tree-in-bud satellites and cavities, but less calcification. Increase lesions had intermediate volumes and tended to contain clusters of nodules rather than individual nodules. The fluctuating patterns were less common. Mix-I-D lesions exhibited significantly more clusters of nodules. Although Mix-D-I lesions showed no statistical differences compared to other types, all these lesions were active, as indicated by cavities and tree-in-bud patterns present in all satellite lesions. When we stratified the Mix-D-I lesions by their pattern of progression, 60% showed in situ expansion, while 40% had new lesions, and those with in situ expansion had significantly more tree-in-bud satellite lesions than any other pattern (Supplementary Table S3).

### 3.3. Longitudinal Characteristics of Lesion Changes May Be Associated with Patient Treatment Outcomes

There were no significant differences between patients with treatment success or treatment failure in demographic or clinical characteristics, nor in most static and dynamic lesions patterns (Table 1, Supplementary Table S2). However, on the initial CT scans, patients with treatment failure showed significantly more tree-in-bud patterns in both primary and satellite lesions, as well as more frequent satellite bronchiectasis (Table 1).

Patients with treatment failure were also significantly more likely to have Mix-D-I lesions than patients successfully treated (25.0% vs. 1.4%, *p* = 0.027), and this tendency persisted in patients with more than two scans, although not reaching statistical significance (50.0% vs. 5.9%, *p* = 0.080). Multivariate analysis confirmed that Mix-D-I lesions were significantly associated with poor treatment outcomes (OR: 17.49, 95% CI: 1.28–239.87, *p* = 0.024). Additionally, treatment failure was significantly associated with the total volume of active lesions on the initial CT scans (OR: 1.71, 95% CI: 0.98–3.01, *p* = 0.041) (Table 1).

## 4. Discussion

This study, which utilized serial CT scans to quantify changes in PTB lesions, revealed extensive heterogeneity in lesion evolution over time. Although diversity was observed in 66.7% of successfully treated patients, it was prominent in 100% of treatment failure cases, particularly regarding changes in lesion volume. We identified five distinct patterns of lesion dynamics, three of which—Decrease, Stable, and Increase—showed similar distributions in both successfully and unsuccessfully treated patients. Mix-D-I lesions, however, characterized by initial regression followed by progression, were associated with treatment failure, as confirmed with multivariable regression analysis. These lesional dynamics observed on serial CT scans could potentially serve as indicators of poor treatment outcomes.

Lesion heterogeneity in PTB patients has been previously characterized [7,27]. In our study, 25.9% of successfully treated patients exhibited the simultaneous presence of lesions with both increased and decreased volumes, whereas all patients who failed treatment had complex multi-pattern lesions. Our study shows that PTB lesions develop in a dynamic, multifaceted process, presumably influenced by the local microenvironmental and immune defenses within the host. The divergent trajectories of different pulmonary lesions likely reflect the interplay of various bacterial and host factors, including heteroresistant *Mtb* subpopulations [16,28], lesion structures that create immune-sheltered zones or drug-poor niches [5,29], microenvironments enriched with mast cells, endothelial cells, fibroblasts, and plasma cells, signaling through type 2 immunity, wound-healing pathways [9] and other elements of the host immune defenses.

Lesion progression and regression were seen in both successfully treated patients and patients with treatment failure, but the pattern of Mix-D-I lesions—characterized by initial regression followed by progression—emerged as a potential indicator of treatment failure. The Mix-D-I pattern generally included cavities and tree-in-bud opacities that have been associated with bronchial spread, increased bacterial proliferation and the development of a more complex microenvironment [29,30,31]. Lesion volume may provide a rough readout of local disease activity influenced by both bacterial burden and host immune responses, and its direction of change may hint at a changing local balance. The Mix-D-I pattern could indicate that anti-TB drugs initially eliminated most actively replicating, drug-sensitive bacilli, leading to reduced lesion volume [32,33], followed by the selective expansion of minor drug-resistant subpopulations or the reactivation of dormant persisters, resulting in subsequent lesion expansion [17,34,35]. Lesion expansion could also be due to structural changes within the lesion that reduced drug penetration, or alternatively, could be the result of immune exhaustion or dysregulation [5,29,36]. We confirmed findings from previous studies that large initial volumes of active lesions, bronchiectasis and tree-in-bud patterns are associated with poor prognosis [37,38,39]. Mix-D-I lesions, which become increasingly apparent with more serial CT scans, were also associated with poor outcomes, but only a minority of our patients had ≥2 CT scans (21/78, 26.9%). Therefore, the association between Mix-D-I lesions and treatment failure needs to be validated in a larger cohort.

Monitoring TB lesion dynamics remains challenging, although PET/CT offers robust quantitative measures for assessing treatment response. These imaging metrics have been shown to correlate with clinical outcomes as early as treatment initiation and one month into therapy, with improved predictive accuracy at six months [12,15]. Several automated segmentation models have been developed using artificial intelligence (AI) [40,41,42], and machine learning can use high-dimensional CT features to identify drug-resistant TB subtypes, assess lesion severity, and predict sputum conversion [43,44,45]. Nijiati et al. found that incorporating follow-up CT scans improved the performance of AI models for predicting PTB outcomes, reaching an AUC of 0.815 [46]. However, due to our small sample size and the complex, heterogeneous nature of PTB lesions that pose challenges for developing and validating AI models, this study relied on traditional manual segmentation and adopted a conservative approach to correct longitudinal data in our limited sample. Lesion volume, a simple yet widely used marker of lung burden, was employed to quantify lesion dynamics and demonstrate its potential predictive value. However, future studies with larger cohorts should investigate additional AI-derived imaging features to improve the modeling of lesion trajectories and assess their utility in predicting treatment outcomes.

Our study had several limitations. The CT scans from patients in this study were collected over varying time intervals, which may have limited our ability to consistently capture lesion evolution patterns. This may also introduce selection bias, since patients with multiple CT scans may represent more severe or closely monitored cases. Second, the lack of detailed treatment data prevented us from correlating the timing of missed doses and CT follow-up time points with lesion dynamics. Additionally, without treatment details, it remains unclear whether the enlargement phase of Mix-D-I lesions occurred during treatment, suggesting drug resistance, or after its completion, suggesting inadequate treatment or reactivation. Furthermore, most of the CT scans came from Belarus, which may have introduced potential biases related to ethnicity and environmental factors. Despite statistical adjustments, the relatively small sample size of our study may have limited the statistical power of our analyses. Lastly, methods for quantifying lesion segmentation and dynamics based on CT scans should be further optimized to reduce operator dependency and enhance usability. Future research may validate lesion-level dynamic patterns in larger, prospectively followed cohorts with standardized imaging and treatment documentation, and integrate imaging with microbiological and host data to better understand mechanisms of treatment failure.

## 5. Conclusions

This is the first study using serial CT scans to quantify the heterogeneity and dynamics of lesions in PTB. The study revealed pervasive diversity in lesion trajectories and identified particular lesion dynamics as potential indicators of poor treatment outcomes. These findings highlight the potential of CT scans for monitoring the response to TB treatment and provide valuable insights for the development of personalized treatment strategies.

## Figures and Tables

**Table 1:** Clinical, CT sampling, lesion integration characteristics and treatment outcome determinants in PTB participants.

	Success (*n* = 70)		Failure (*n* = 8)		*p*	Univariable		Multivariable	
						OR (95%CI)	*p*	OR (95%CI)	*p*
Demographic &. Clinical									
Age, years									
≥35	48	(68.6%)	5	(62.5%)	0.706	0.73 (0.17–3.05)	0.668	-	
Male	37	(52.9%)	6	(75.0%)	0.285	2.32 (0.50–10.74)	0.261	-	
Patient-Level Lesion Static Data									
Baseline total lesion									
Baseline total lesion number	2.0	(2.0–3.0)	2.5	(2.0–3.3)	0.348	1.17 (0.94–1.45)	0.188		
Baseline total lesion volume (cm^3^) *	7.6	(2.8–31.2)	29.0	(20.3–106.1)	0.091	1.75 (1.06–2.89)	0.021		
Baseline total active lesion									
Baseline total active lesion number	2.0	(1.0–2.8)	2.0	(1.8–3.0)	0.478	1.27 (0.87–1.86)	0.239		
Baseline total active lesion volume (cm^3^) *	6.6	(2.4–31.2)	28.9	(20.3–106.2)	0.071	1.76 (1.06–2.92)	0.019	1.71 (0.98—3.01)	0.041
The presence of cavities at baseline	33	(47.1%)	5	(62.5%)	0.476	1.76 (0.43–7.27)	0.430		
The number of cavities at baseline	0.0	(0.0–1.0)	1.0	(0.0–1.3)	0.291	1.63 (0.81–3.27)	0.194		
The presence of a specific primary lesion type									
Consolidation	31	(44.3%)	6	(75.0%)	0.141	3.26 (0.70–15.09)	0.111		
Nodule	39	(55.7%)	4	(50.0%)	1.000	0.80 (0.20–3.20)	0.749		
Cluster of nodules	30	(42.9%)	3	(37.5%)	1.000	0.85 (0.20–3.50)	0.815		
Tree in buds	0	(0.0%)	2	(25.0%)	0.009	54.23 (2.35–1254.09)	0.003		
Strand	4	(5.7%)	0	(0.0%)	1.000	0.87 (0.04–17.60)	0.926		
Atelectasis	1	(1.4%)	0	(0.0%)	1.000	2.73 (0.10–72.36)	0.582		
Presence of satellite lesion	64	(91.4%)	6	(75.0%)	0.188	0.26 (0.05–1.39)	0.141		
The presence of a specific satellite lesion type									
Bronchiectasis	1	(1.4%)	2	(25.0%)	0.027	17.82 (2.02–157.34)	0.010		
Tree in buds	14	(20.0%)	5	(62.5%)	0.018	6.12 (1.42–26.34)	0.014		
Cluster of nodules	1	(1.4%)	0	(0.0%)	1.000	2.73 (0.10–72.36)	0.582		
Reversed halo sign	1	(1.4%)	0	(0.0%)	1.000	2.73 (0.10–72.36)	0.582		
Strand	1	(1.4%)	0	(0.0%)	1.000	2.73 (0.10–72.36)	0.582		
The presence of a specific accompanying characteristics type									
Calcification	14	(20.0%)	1	(12.5%)	1.000	0.78 (0.12–4.93)	0.787		
Cavity	33	(47.1%)	5	(62.5%)	0.476	1.76 (0.43–7.27)	0.430		
Slight to moderate density	52	(74.3%)	5	(62.5%)	0.675	0.55 (0.13–2.34)	0.431		
Fibrosis	4	(5.7%)	0	(0.0%)	1.000	0.87 (0.04–17.60)	0.926		
Patient-Level Lesion Dynamic Data									
New lesion									
The presence of newly emerged lesion	14.0	(20.0%)	4	(50.0%)	0.078	3.90 (0.93–16.26)	0.067		
The number of newly emerged lesion	0.0	(0.0–0.0)	0.5	(0.0–1.0)	0.066	1.38 (0.96–1.98)	0.111		
The presence of a specific volume change pattern									
Decrease	41	(58.6%)	7	(87.5%)	0.143	3.55 (0.58–21.84)	0.128		
Mix-D-I (*n* = 21)	1	(5.9%)	2	(50.0%)	0.080	11.00 (0.98–123.98)	0.046	17.49 (1.28—239.87)	0.024
Stable	36	(51.4%)	5	(62.5%)	0.715	1.49 (0.36–6.14)	0.581		
Increase	29	(41.4%)	4	(50.0%)	0.716	1.41 (0.35–5.64)	0.631		
Mix-I-D (*n* = 21)	3	(17.6%)	1	(25.0%)	1.000	1.78 (0.19–16.69)	0.622		
CT series									
Number of CT scans per person	2.0	(2.0–2.0)	2.5	(2.0–3.0)	0.207				
Patients with more than 2 CT scans	17	(24.3%)	4	(50.0%)	0.201				

Data are median (IQR), mean (SD) or *n* (%). * Lesion volume was standardized using *Z*-scores (mean = 0, SD = 1) in both univariable and multivariable analyses.

## Data Availability

All CT scan data used in this study were retrieved from the NIAID TB Portals Program (https://tbportals.niaid.nih.gov/). The associated de-identified clinical metadata, including treatment history and outcomes, were also obtained from this publicly accessible database.

## Supplementary Materials

The following supporting information can be downloaded at: https://www.mdpi.com/article/10.3390/diagnostics16060892/s1, Supplementary Text S1: Independent lesion delineation criteria; Supplementary Text S2: Annotation rules for lesion imaging characteristics; Supplementary Text S3: Estimation scheme for the volume measurement errors derived from manual segmentation; Supplementary Text S4: Patient-Level Lesion Imaging Parameters; Supplementary Table S1: Country distribution of cases from the TB portals database and longitudinal retrospective study; Supplementary Table S2. Clinical, Lesion Integration Characteristics and Treatment Outcome Determinants in PTB Participants; Supplementary Table S3. The baseline characteristics of lesions with different volume evolution patterns; Supplementary Table S4: Comprehensive longitudinal lesion-level data for all 125 patients; Supplementary Figure S1. Selection scheme for the study population and CT scans; Supplementary Figure S2. CT segmentation and lesion annotations; Supplementary Figure S3. Estimation scheme for the measurement error range of volumes derived from manual segmentations; Supplementary Figure S4. The composition of different volume change patterns of lesions within 47 PTB patients.
