# Supplementary material for: Divergent Evolution of Tuberculosis Lesions During Treatment: A Longitudinal CT-Based Analysis of Progression and Regression Patterns
Source: Diagnostics (Basel). 2026 Mar 18;16(6):892. doi: 10.3390/diagnostics16060892 (PMC13025260; doi:10.3390/diagnostics16060892)
Supplement: Supplementary file 1 [file diagnostics-16-00892-s001.zip › diagnostics-3978803-supplementary.pdf]

Supplementary Text S1: Independent lesion delineation criteria

We manually delineated the lesions in all lung CT scans collected from pulmonary tuberculosis (TB) patients. For each lesion, we identified individual lesions and defined VOI (volume of interest) based on the following criteria:

1） Completely separated lesions that clearly originate from different small bronchi were defined as separate VOIs.

2） Lesion groups with minor spatial separation, regardless of differing radiological features, but within the same small bronchial drainage area and lacking clear boundaries, were treated as a single VOI.

3） Lesion groups with minor spatial separation but without certainty of being in the same bronchial drainage area, and in close proximity to each other, were also treated as a single VOI. For example, if small nodules are present around a consolidation area, they were considered part of the same VOI, assumed to be satellite lesions resulting from the primary lesion's bronchial spread.

4） Partially or completely fused lesions, regardless of differences in radiological features, were considered a single VOI.

5） Clusters of nodules were defined as multiple nodules densely distributed in a specific bronchial drainage area and treated as a single VOI without distinguishing each individual nodule.

Supplementary Text S2: Annotation rules for lesion imaging characteristics

For each independent lesion VOI in the patient's lungs, three categories of imaging features were added to characterize the lesion's radiological characteristics:

1） **Primary lesion type**: This refers to the predominant morphological feature within the VOI, representing the main lesion type ("Atelectasis", "Cluster of Nodules", "Consolidation", "Nodule", "Strand", "Tree-in-buds").

2） **Satellite lesion type**: These are additional morphological features surrounding the primary lesion but exhibiting varying degrees of fusion with it. The VOI may have no satellite lesions, recorded as "none" ("None", "Cluster of Nodules", "Reversed Halo Sign", "Tree-in-buds", "Bronchiectasis", "Strand").

3） **Accompanying characteristics type**: These features are defined based on density and activity, including ("Slight to Moderate Density", "Cavity", "Fibrosis", "Calcification"). "Calcification" and "Fibrosis" represent proliferative lesions, while "Cavity" and "Slight to Moderate Density" lesions represent exudative lesions. Generally, lesions may undergo resolution, leading to "Fibrosis" and "Calcification", indicating strong host immune activity or effective anti-TB treatment. Conversely, lesions may progress, showing signs of caseous necrosis and cavity formation. Lesions exhibiting exudative features ("Slight to Moderate Density" and "Cavity") were categorized as "Active" lesions.

Supplementary Text S3: Estimation scheme for the volume measurement errors derived from manual segmentation

When manually segmenting the same lesion at different time points for a single patient, volume measurement errors may arise. In this study, we assumed that the volume measurement error for lesions segmented by experienced clinicians falls within a spatial range defined by expanding or contracting the lesion's VOI by one voxel in all directions. Since lesion VOIs in CT images are composed of irregular voxel stacks, directly calculating volume errors based on the VOI is complex. To simplify, we approximated each lesion VOI as a sphere with equivalent volume, referred to as the sphere-VOI. The volume error range was then estimated by calculating the change in volume resulting from a small increase or decrease in the sphere-VOI's diameter (Supplementary Figure 3A).

To standardize, all CT voxel dimensions were converted to equivalent spheres. The median sphere-voxel diameter in the TB Portals database, accounting for differences in scanner types and specifications, was determined to be 1.33 mm. For any given lesion volume V, the $percentage\ reduction\ in\ volume\ (\%)$ can be estimated based on：

$$percentage\ reduction\ in\ volume\ (\%) = 100 \times \left(1 - \left(\frac{\left(\frac{3V}{4\pi}\right)^{\frac{1}{3}} - 1.33}{\frac{3V}{4\pi}}\right)^{3}\right)$$

Simulation calculations show that larger lesion volumes result in smaller measurement errors, while smaller lesions exhibit larger measurement errors under the same resolution. Therefore, we established multiple volume change percentage thresholds to define the error range. In practice, for a given lesion VOI volume, the measured volume will fall into different volume intervals, each associated with a corresponding measurement error percentage threshold (Supplementary Figure 3B-C). For example, if a lesion has a baseline volume of 600 mm^3, it falls within the 500 mm^3-1000 mm^3 range, corresponding to a measurement error percentage threshold of 55%. Thus, if, in the next CT scan, the absolute volume change between the current and baseline measurements is below 55%, the lesion is considered not to have undergone significant volume change, with the difference attributed to measurement error. If the change exceeds this threshold, the lesion is considered to have undergone substantial enlargement or reduction.

Supplementary Text S4: Patient-Level Lesion Imaging Parameters

We categorized all lesion-level volume and imaging annotation parameters into two types: static data and dynamic data. The former refers to lesion information at the patient's baseline CT scan, while the latter encompasses longitudinal data that tracks lesion changes over time. Additionally, since multiple independent lesions may exist, we integrated the following lesion information at the patient level. It is important to note that for any variables requiring the calculation of proportions, the denominator is the total number of lesions within the patient.

1） **Static Integrated Data**:
   a) Baseline total lesion number and volume.
   b) Baseline total active lesion number and volume.
   c) The presence and number of cavities at baseline.
   d) The presence and proportion of a specific morphological feature in primary lesions at baseline.
   e) The presence and proportion of a specific morphological feature in satellite lesions at baseline.
   f) The presence and proportion of a specific feature in accompanying characteristics lesions at baseline.

2） **Dynamic Integrated Data**:
   a) The presence and number of newly emerged lesions relative to baseline.
   b) The presence and proportion of a specific lesion volume change pattern.

Supplementary Table S1: Country distribution of cases from the TB portals database and longitudinal retrospective study

Country	All cases in NIAID TB portals (n=1456)		Case included in longitudinal retrospective study (n=125)	
Belarus	998	(68.5%)	115	(91.3%)
Romania	299	(20.5%)	7	(5.6%)
Azerbaijan	82	(5.6%)	1	(0.8%)
Ukraine	29	(2.0%)	0	(0.0%)
Moldova	17	(1.2%)	0	(0.0%)
Georgia	14	(1.0%)	1	(0.8%)
China	11	(0.8%)	1	(0.8%)
India	5	(0.3%)	0	(0.0%)
Kazakhstan	1	(0.1%)	0	(0.0%)

Supplementary Table S2. Clinical, Lesion Integration Characteristics and Treatment Outcome Determinants in PTB Participants.

	SUCCESS (n=70)		FAILURE (n=8)		*P*	Univariable	
						OR(95%CI)	*P*
**Demographic &. Clinical**							
**Age**							
⩾35y	48	(68.6%)	5	(62.5%)	0.706	0.73(0.17-3.05)	0.668
**Male**	37	(52.9%)	6	(75.0%)	0.285	2.32(0.50-10.74)	0.261
**Country**							
China	1	(1.4%)	0	(0.0%)	1.000	ref	-
Belarus	66	(94.3%)	8	(100.0%)	1.000	0.38(0.01-10.18)	0.597
Georgia	1	(1.4%)	0	(0.0%)	1.000	1.00(0.01-92.43)	1.000
Romania	2	(2.9%)	0	(0.0%)	1.000	0.60(0.01-49.45)	0.821
**Education**							
Basicschool	0	(0.0%)	1	(12.5%)	0.103	ref	
Complete school	17	(24.3%)	2	(25.0%)	1.000	0.05(0.00-1.52)	0.060
College (bachelor)	33	(47.1%)	5	(62.5%)	0.476	0.05(0.00-1.52)	0.062
Higher (university)	18	(25.7%)	0	(0.0%)	0.187	0.01(0.00-0.64)	0.012
Not Reported	2	(2.9%)	0	(0.0%)	1.000	0.07(0.00-5.49)	0.182
**Employment**							
Disabled	6	(8.6%)	1	(12.5%)	0.546	ref	
Retired	10	(14.3%)	2	(25.0%)	0.601	1.03(0.11-9.77)	0.978
Unemployed	17	(24.3%)	3	(37.5%)	0.416	0.87(0.10-7.18)	0.895
Student	3	(4.3%)	0	(0.0%)	1.000	0.62(0.02-19.59)	0.780
Employed	33	(47.1%)	2	(25.0%)	0.285	0.32(0.04-2.89)	0.335
Not Reported	1	(1.4%)	0	(0.0%)	1.000	1.44(0.04-56.14)	0.847
**TB type**							
PTB	54	(77.1%)	4	(50.0%)	0.193	ref	
PTB &. EPTB	16	(22.9%)	4	(50.0%)	0.193	3.30(0.80-13.64)	0.104
**Type of resistance**							
Sensitive	14	(20.0%)	1	(12.5%)	1.000	ref	
Mono	5	(7.1%)	1	(12.5%)	0.490	2.64(0.22-31.07)	0.446
MDR	35	(50.0%)	2	(25.0%)	0.268	0.68(0.08-5.64)	0.726
Pre-XDR	3	(4.3%)	0	(0.0%)	1.000	1.38(0.05-41.67)	0.856
XDR	13	(18.6%)	4	(50.0%)	0.063	3.22(0.44-23.60)	0.223
**BMI**	22.0	(20.0-24.0)	21.4	(19.0-22.6)	0.494	0.94(0.75-1.17)	0.577
**Comorbidity**							
HIV	7	(10.0%)	1	(12.5%)	1.000	1.69(0.25-11.43)	0.604
Diabetes	4	(5.7%)	0	(0.0%)	1.000	0.87(0.04-17.60)	0.926
Hepatitis C	7	(10.0%)	0	(0.0%)	1.000	0.50(0.03-9.52)	0.613
Post COVID-19	1	(1.4%)	0	(0.0%)	1.000	2.73(0.10-72.36)	0.582
Renaldisease	1	(1.4%)	0	(0.0%)	1.000	2.73(0.10-72.36)	0.582
Cytostatics	1	(1.4%)	0	(0.0%)	1.000	2.73(0.10-72.36)	0.582
Systemically administered glucocorticoids	1	(1.4%)	0	(0.0%)	1.000	2.73(0.10-72.36)	0.582
TNF-α antagonists	1	(1.4%)	0	(0.0%)	1.000	2.73(0.10-72.36)	0.582
Psychiatricillness	1	(1.4%)	0	(0.0%)	1.000	2.73(0.10-72.36)	0.582
Others	22	(31.4%)	5	(62.5%)	0.117	3.39(0.81-14.16)	0.090
Not specified	4	(5.7%)	1	(12.5%)	0.427	2.96(0.40-21.76)	0.323
**Patient-Level Lesion Static Data**							
**The proportion of a specific primary lesion type (%)**							
Consolidation	68.3	(30.7)	56.2	(36.9)	0.400	1.00(0.96-1.02)	0.403
Nodule	68.7	(27.2)	66.7	(11.8)	0.884	1.00(0.96-1.03)	0.877
Cluster of nodules	68.4	(31.3)	37.5	(21.7)	0.107	0.97(0.93-1.01)	0.127
Tree in buds	-	(-)	41.7	(11.8)	-	-	-
Strand	25.4	(11.0)	-	(-)	-	-	-
Atelectasis	50.0	(-)	-	(-)	-	-	-
**The proportion of a specific satellite lesion type (%)**							
Bronchiectasis	33.0	(-)	18.8	(8.8)	0.407	0.89(0.69-1.14)	0.312
Tree in buds	55.6	(26.9)	57.5	(41.1)	0.907	1.00(0.97-1.03)	0.873
Cluster of nodules	50.0	(-)	-	(-)	-	-	-
Reversed halo sign	50.0	(-)	-	(-)	-	-	-
Strand	50.0	(-)	-	(-)	-	-	-
**The proportion of a specific accompanying characteristics type (%)**							
Calcification	52.5	(32.2)	25.0	(-)	0.423	0.99(0.93-1.04)	0.599
Cavity	63.0	(29.5)	66.7	(35.8)	0.804	1.00(0.97-1.03)	0.803
Slight to moderate density	78.5	(26.9)	88.3	(16.2)	0.428	1.01(0.98-1.05)	0.520
Fibrosis	25.4	(11.0)	-	(-)	-	-	-
**Patient-Level Lesion Dynamic Data**							
**The proportion of a specific volume change pattern (%)**							
Decrease	64.0	(29.6)	50.7	(15.1)	0.252	0.98(0.96-1.01)	0.267
Mix-D-I (n=21)	40.0	(-)	19.6	(7.6)	0.272	0.89(0.72-1.10)	0.232
Stable	68.2	(26.9)	38.8	(11.0)	0.021	0.95(0.91-1.00	0.023
Increase	60.6	(30.0)	40.5	(11.2)	0.198	0.98(0.94-1.02)	0.230
Mix-I-D (n=21)	41.1	(8.4)	50.0	(-)	0.456	1.11(0.85-1.45)	0.436

*Data are median (IQR), mean (SD) or n (%). *Lesion volume was standardized using Z-scores (mean = 0, SD = 1) in univariable analyses.*

Supplementary Table S3. The baseline characteristics of lesions with different volume evolution patterns.

	Stable (n = 84)	P	Decrease (n = 85)	P	Increase (n = 28)	P	Mix-I-D (n = 5)	P	Mix-D-I (n = 5)	P	Mix-D-I in situ expansion (n = 3)	P	Mix-D-I new lesions (n = 2)	P
**Volume (cm3), median (IQR)**	0.71 (0.28-2.50)	0.002	3.74 (0.76-20.38)	< 0.001	1.73 (0.68-5.39)	0.011	2.64 (0.74-3.10)	0.853	0.71 (0.37-1.93)	0.404	0.71 (0.54-5.18)	0.789	1.02 (0.56-1.47)	0.328
**Primary lesion**														
Atelectasis	0 (-)	-	1 (1.2)	-	0 (-)	-	0 (-)	-	0 (-)	-	0 (-)	-	0 (-)	-
Cluster of Nodules	15 (17.9)	0.144	16 (18.8)	0.229	13 (46.4)	0.005	4 (80.0)	0.012	1 (20.0)	1.000	0 (-)	-	1 (50.0)	0.418
Consolidation	14 (16.7)	0.009	34 (40.0)	< 0.001	7 (25.0)	0.973	1 (20.0)	1.000	0 (-)	-	0 (-)	-	0 (-)	-
Nodule	51 (60.7)	< 0.001	32 (37.6)	0.084	7 (25.0)	0.033	0 (-)	-	4 (80.0)	0.179	3 (100.0)	0.092	1 (50.0)	1.000
Strand	2 (2.4)	1.000	2 (2.4)	1.000	1 (3.6)	0.520	0 (-)	-	0 (-)	-	0 (-)	-	0 (-)	-
Tree-in-buds	2 (2.4)	-	0 (-)	-	0 (-)	-	0 (-)	-	0 (-)	-	0 (-)	-	0 (-)	-
**Satellite lesion**														
Cluster of Nodules	1 (1.2)	-	0 (-)	-	0 (-)	-	0 (-)	-	0 (-)	-	0 (-)	-	0 (-)	-
Reversed halo sign	0 (-)	-	0 (-)	-	0 (-)	-	1 (20.0)	-	0 (-)	-	0 (-)	-	0 (-)	-
Tree-in-buds	4 (4.8)	0.029	15 (17.6)	0.023	1 (3.6)	0.327	1 (20.0)	0.449	2 (40.0)	0.096	2 (66.7)	0.033	0 (-)	-
Bronchiectasis	0 (-)	-	2 (2.4)	0.569	1 (3.6)	0.355	0 (-)	-	0 (-)	-	0 (-)	-	0 (-)	-
Strand	0 (-)	-	0 (-)	-	1 (3.6)	-	0 (-)	-	0 (-)	-	0 (-)	-	0 (-)	-
none	79 (94.0)	0.011	68 (80.0)	0.062	25 (89.3)	0.773	3 (60.0)	0.145	3 (60.0)	0.145	1 (33.3)	0.052	2 (100.0)	1.000
**Accompanying characteristics**														
Slight to moderate density	55 (65.5)	0.530	49 (57.7)	0.312	17 (60.7)	1.000	4 (80.0)	0.652	4 (80.0)	0.652	2 (66.7)	1.000	2 (100.0)	0.528
Cavity	12 (14.3)	0.007	30 (35.3)	0.005	7 (25.0)	1.000	1 (20.0)	1.000	1 (20.0)	1.000	1 (33.3)	0.574	1 (20.0)	1.000
Fibrosis	2 (2.4)	1.000	2 (2.4)	1.000	1 (3.6)	0.520	0 (-)	-	0 (-)	-	0 (-)	-	0 (-)	-
Calcification	15 (17.9)	0.010	4 (4.7)	0.038	3 (10.7)	1.000	0 (-)	-	0 (-)	-	0 (-)	-	0 (-)	-

The above analysis was based on the remaining 207 lesions, excluding the 27 that appeared only in the final scan.

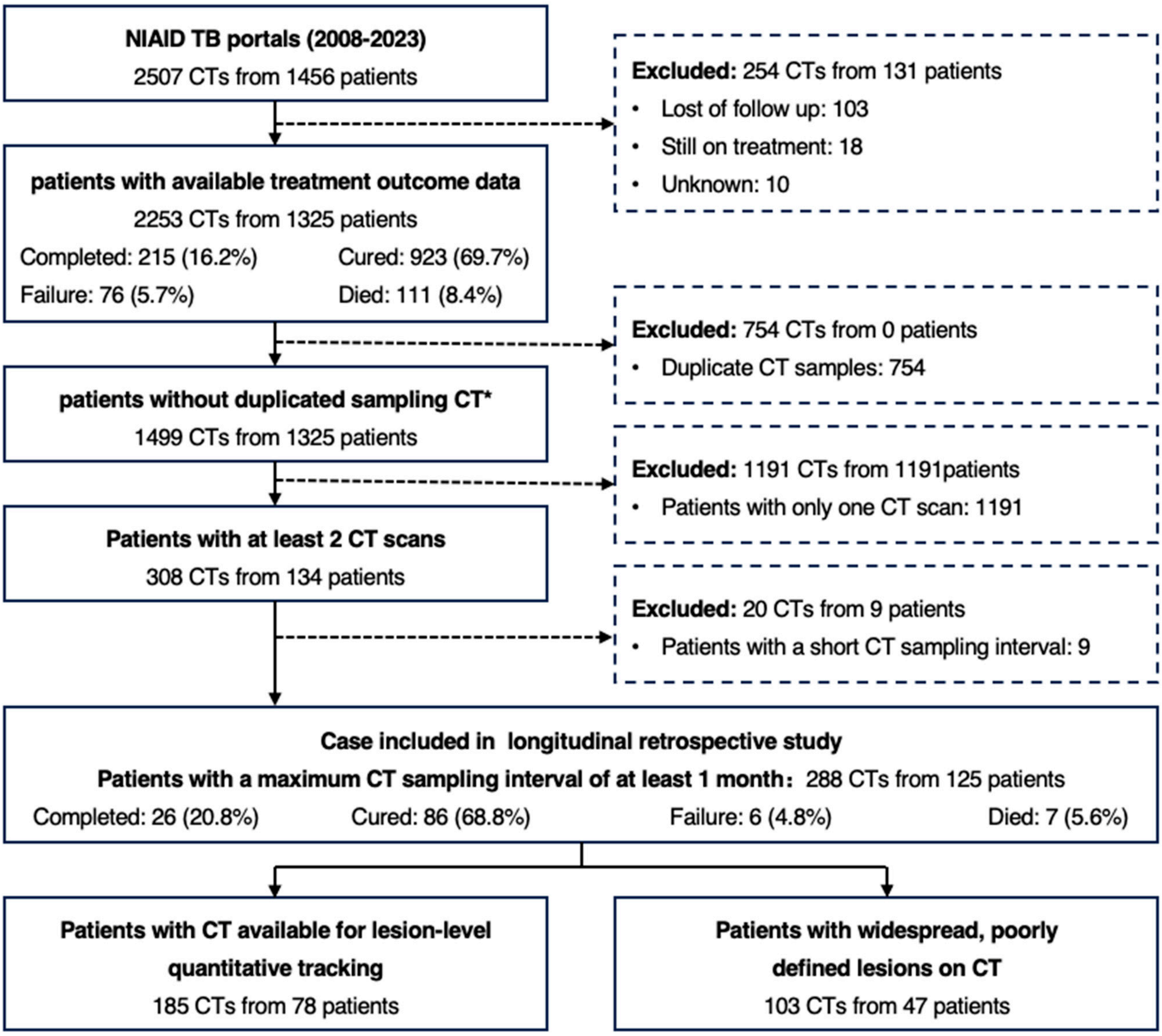

Supplementary Figure S1. Selection scheme for the study population and CT scans. * Duplicated sampling CT is defined as cases where multiple CT images were retrieved on the same day.

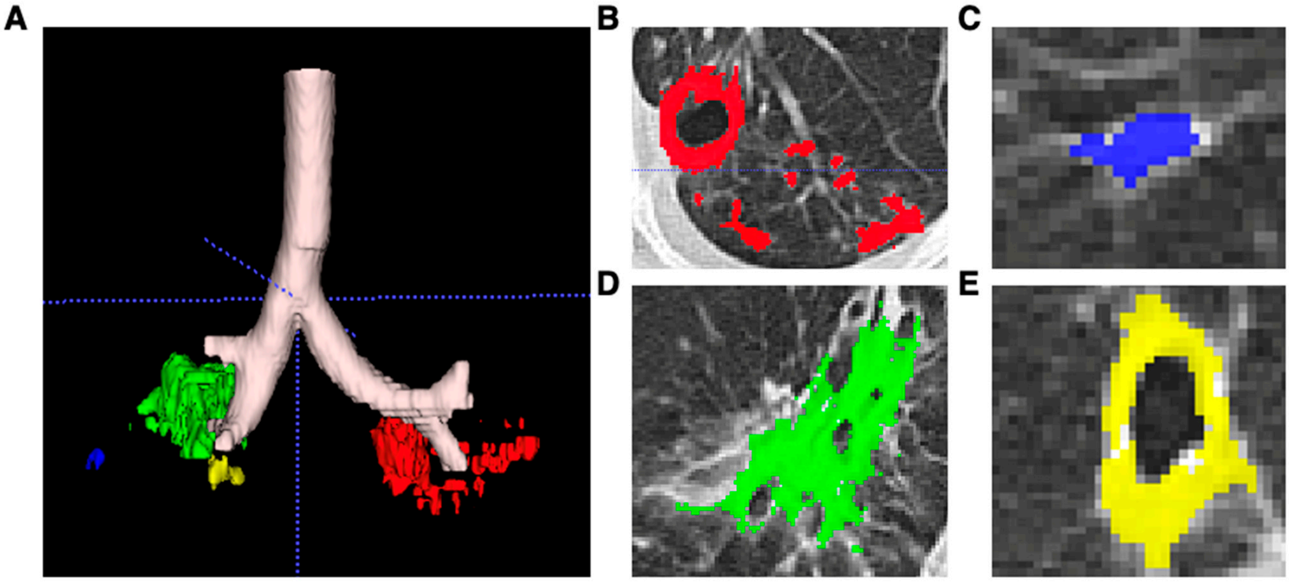

Supplementary Figure S2. CT segmentation and lesion annotations. (A) Each lesion in the CT scans was independently segmented to extract lesion-level characteristics, including volume measurements, morphological features, and dynamic evolution patterns. The three-dimensional reconstruction model of all lesions in a patient is shown, with bronchial structures as a reference (light pink), and individual lesions differentiated by distinct colors. **(B)** An independent lesion (red), with consolidation as the primary lesion and a cluster of nodules as the surrounding satellite lesions, accompanied by a cavity feature. **(C)** An independent lesion (blue), with a nodule as the primary lesion, no satellite lesions, and an accompanying characteristic of slight to moderate density. **(D)** An independent lesion (green), with consolidation as the primary lesion, no satellite lesions, and fibrosis as the accompanying feature. **(E)** An independent lesion (yellow), with a nodule as the primary lesion, no satellite lesions, and a cavity as the accompanying characteristic.

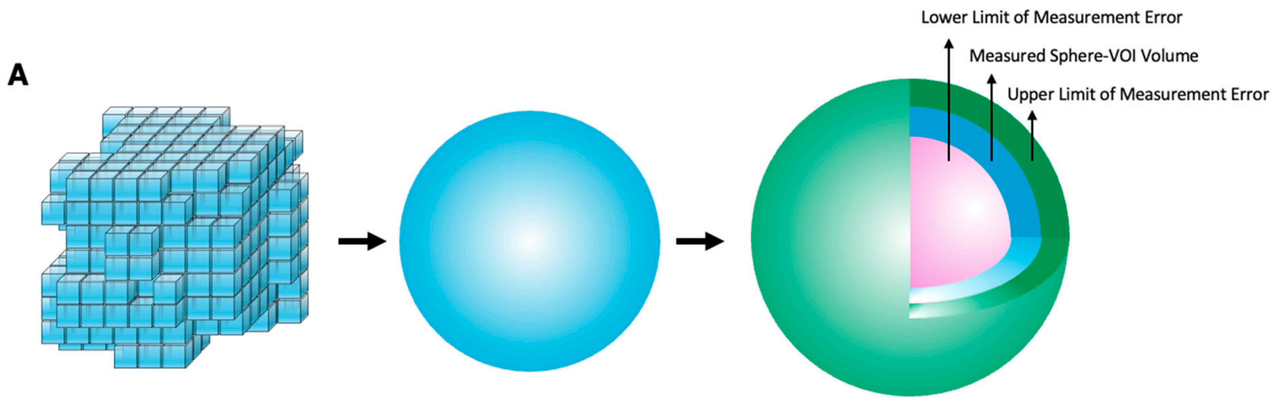

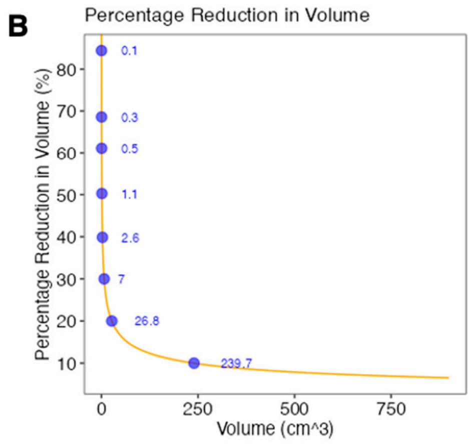

C

lower volume interval	upper volume interval	threshold (%)
0	100	85
100	300	75
300	500	65
500	1000	55
1000	2500	45
2500	7000	35
7000	25000	25
25000	250000	15
250000	800000	5

Supplementary Figure S3. Estimation scheme for the measurement error range of volumes derived from manual segmentations. (A) The volume of interest (VOI) for each lesion (the irregular blue shape) is approximated as a sphere-VOI (blue spheres) with an equivalent volume. The range of lesion volume measurement error is estimated by calculating the volume change caused by the increase or decrease of the sphere-VOI's diameter (right-side pink sphere indicates the lower limit of the lesion volume error, while the green sphere represents the upper limit). **(B)** A fitting curve showing the spatial reduction ratio produced by shrinking a voxel (1.33mm) inward for any given lesion VOI volume. The X-axis represents the lesion VOI volume, and the Y-axis shows the percentage of spatial volume reduction. The points in the graph correspond to multiple volume change percentage threshold points set in this study. **(C)** A comparison table of the volume error range defined by multiple volume change percentage thresholds. For a given lesion VOI volume, its volume will fall within a specific range. If the volume difference between two consecutive measurements of the same lesion VOI falls within this threshold, it is considered that the volume has not changed, and the difference is attributed to measurement error. If the volume difference exceeds the threshold, the lesion is considered to have undergone significant enlargement or reduction.

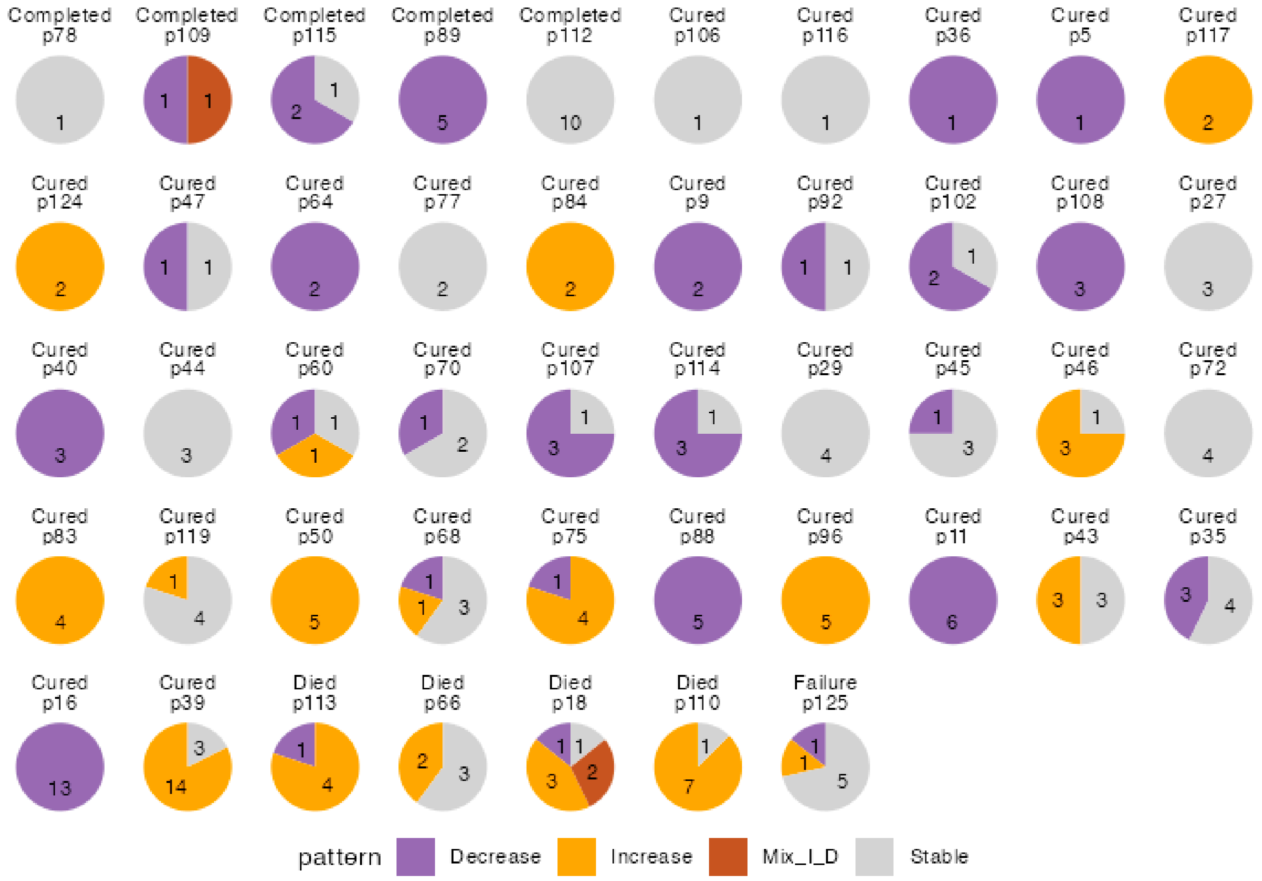

Supplementary Figure S4. The composition of different volume change patterns of lesions within 47 PTB patients. Due to poorly defined lesion boundaries, lesion-level quantitative tracking was not conducted for these 47 patients, and the changes in lesion volume were recorded by experienced clinicians. There were 42 treatment success patients and 5 treatment failure patients, with 5 patients having a single lesion (all treatment successes). Among the multi-lesion patients, 45.9% (17/37) treatment success patients exhibited two or more distinct volume change patterns, while all treatment failure patients displayed coexisting multiple volume change patterns. Different colors represent the different volume change patterns.
